# Effects of aerobic exercise of different intensities on the social, emotional, and financial functioning of healthy older adults: results from a 16-week exercise randomized control trial

**DOI:** 10.1007/s11357-025-01655-0

**Published:** 2025-04-21

**Authors:** Charleen J. Gust, Renée Martin-Willett, Laurel P. Gibson, Gregory Giordano, Douglas R. Seals, Angela D. Bryan

**Affiliations:** Department of Psychology & Neuroscience, University of Colorado Boulder, Boulder, CO USA

**Keywords:** Physical activity, Exercise, Aging, Functional ability

## Abstract

Better social, emotional, and financial functioning are associated with improved health outcomes in older adults. While the literature supports a positive relationship between physical activity and increased functional ability in older adulthood, the intensity of physical activity necessary to achieve these gains remains uncertain. To address this gap, the current analysis uses data collected as part of a larger randomized controlled trial to examine the effects of a supervised exercise intervention on measures of social, emotional, and financial functioning. Healthy older adults (*n* = 211) completed baseline assessments including cardiovascular fitness and health and functioning measures and were randomly assigned to 16 weeks of either: (1) low intensity continuous training (LICT); or (2) moderate intensity continuous training + interval training (MICT + IT). Participants across exercise conditions reported a significant decrease from baseline to post-intervention in depression [*F*(1, 158) = 22.17, *p* < .001] and instrumental risk taking [*F*(1, 157) = 6.72, *p* < .05]. Further, participants assigned to the MICT + IT condition showed significantly greater improvements in loneliness relative to those in the LICT condition [*F*(1, 157) = 5.99, *p* < .05]. By and large, healthy older adults experienced some improvements in functional ability after 16 weeks of exercise. Though most changes were not related to the intensity of exercise condition, associations between increased cardiovascular fitness and both depression and anxiety suggest that exercising at an intensity that improves fitness may confer greater benefits in some domains.

Trial registration: ClinicalTrials.gov, NCT02068612.

## Introduction

By 2030, over 16% of the global population will be older adults, thus diverse sectors including research have committed to measurable acts aimed at improving the lives of older adults [1]* – 2030*, 2021). Meanwhile, meta-analytic analyses suggest habitual physical activity or exercise is one promising area that may contribute to health and wellbeing for older adults [2–4].

While exercise has been well documented as a modifiable behavior associated with physical wellness, less is known as to what the optimum intensity should be. A recent systematic review [5] showed that high intensity interval training (HIIT) provided comparable or greater cardiorespiratory fitness benefits to older adults than moderate intensity continuous training (MICT), while Lee et al. [6] reported mortality levels were lower when individuals engaged in more moderate and vigorous exercise versus low intensity.

A focus on quality of life, rather than quantity of life as is measured with mortality, requires explication of the domains that impact older adult reports of higher satisfaction and independence. The concept of instrumental activities of daily living (IADLs) goes beyond basic tasks of daily life such as dressing oneself and toileting to include more complicated tasks that require complex thought, higher order cognitive function, and organization such as managing finances and social interactions [7]. IADLs are strongly associated with emotional functioning [8], and both emotional functioning and IADLs are predictive of cognitive decline in aging (A. [9]) While there is some evidence that lifestyle activity decreases IADL disability [10] evidence for the relationship of exercise intensity to improvements in specific components of function that contribute to greater IADL capacity is more scarce and mixed.

For example, studies report conflicting effects of physical activity intensity on levels of social isolation or loneliness [11]. One systematic review of only low-intensity interventions (i.e., with no comparison to higher intensity activities) reported an overall benefit to depression [12], while another broader review on physical activity and affective response was equivocal [13]. Finally, to our knowledge, there are no studies to date that compare the effects of exercise at different levels of intensity on financial functioning. Overall, though there is little academic research in this domain, this is an issue of critical practical importance to the older adult population as evidenced by extensive coverage from respected older adult advocacy organizations such as AARP [14], Consumer Reports [15], and the American Bar Association [16], as well as potential impacts on functioning and IADLs that are associated with higher quality of life [17].

Accordingly, in the present study we conducted a comparative effectiveness randomized control trial of moderate intensity continuous training plus high intensity intervals (MICT + IT) and low intensity continuous training (LICT) on measures of emotional, social, and financial functioning in a large sample of healthy older adults. First, we examined associations between baseline volume of exercise behavior (measured by minutes of moderate-to-vigorous exercise per week) and functioning. We then tested whether exercise intensity condition had differential effects on function. Because of individual variability in adaptation to exercise [18], we also examined associations between change from pre- to post-intervention in cardiopulmonary fitness (VO_2_peak) and functional outcomes.

## Methods

### Participants

**Fig. 1:**
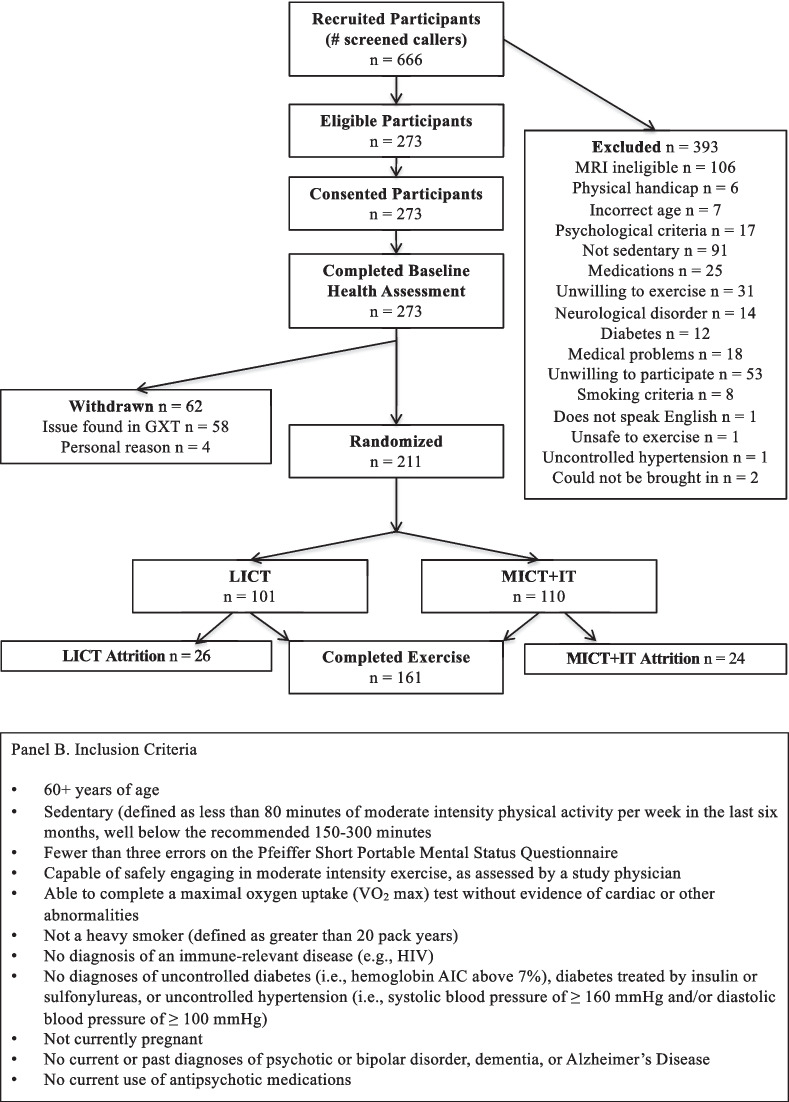
CONSORT diagram and inclusion criteria

This study was approved by the University of Colorado Institutional Review Board and carried out in accordance with the Declaration of Helsinki. The data analyzed here were collected as part of a pre-registered randomized controlled trial aimed at understanding whether exercise can improve social, emotional, and economic functioning in older adults and whether those improvements are linked to changes in neurocognitive structure and function (Clinicaltrials.gov NCT02068612). Participants were recruited from the greater Denver Boulder area between April 2014 and November 2018 using Craigslist, social media advertisements, ResearchMatch, university listservs, mailed flyers, and flyers posted in community spaces.^1^ Participants were compensated up to $300. Eligibility screening was completed via telephone by trained research staff. Inclusion criteria are displayed in Fig. 1B.

### Procedure (see Appendix Table 4)

#### Baseline

After providing written informed consent, all participants completed a baseline health assessment, including a medical evaluation and a physician-supervised graded exercise test to ensure older adults could safely perform a VO2peak test. Participants without abnormal cardiovascular responses to the physician-supervised graded exercise test then completed a second graded exercise test with measurement of oxygen consumption (VO_2_peak), a demographics questionnaire, and measures of emotional, social, and financial functioning on a separate day. The baseline graded exercise tests were performed an average of 18 days apart. All data collection took place at University of Colorado Boulder.

#### Exercise intervention

A random number generator was used by study staff to generate a separate random assignment table for each gender. A research assistant then block-randomized participants by gender to either the LICT or MICT + IT condition. As this was a behavioral trial, it was not possible for participants to be fully blind to condition. Rather, they were blind to study hypotheses as well as the exact nature of the exercise condition to which they were not assigned. All participants were assigned to 16 weeks of supervised exercise three times per week at an exercise laboratory on the University of Colorado Boulder campus. Most participants exercised on a treadmill, however, one participant with orthopedic limitations and balance concerns exercised on a cycle ergometer.

Sessions were supervised by trained research staff and were approximately 30 min long. Participants in the LICT condition were instructed to exercise at 50% of their maximum heart rate (HRmax; previously determined by taking the highest heart rate achieved during the two graded exercise tests). In the MICT + IT condition, participants’ target heart rate and interval training frequency progressively increased throughout the intervention (e.g., participants exercised at 60% of HRmax with no interval training during weeks 1–3; 75–80% of HRmax with one session per week including interval training during weeks 4–6; etc.). Interval training consisted of 3 × 3 min intervals at 85–95% of HRmax. Participants were fitted with a Polar T31 Heart Rate Monitor (Polar Electro, Inc, Woodbury, NY; [19]) during sessions to assess adherence. At the end of the 16-week exercise intervention, participants completed a second graded exercise test and measures of exercise behavior and emotional, social, and financial functioning. Our intervention conditions were directly drawn from previous exercise studies conducted with older adults (e.g., [20]).

### Measures

#### Demographics

Participants reported their age, sex, race/ethnicity, education, and employment status. Direct measurements of height and weight were collected to calculate body mass index (BMI).

#### Exercise behavior

Minutes of moderate-to-vigorous intensity exercise during the previous 7 days was assessed using the interviewer-guided Stanford Seven-Day Physical Activity Recall (PAR) [21].

#### Emotional functioning

##### Depression

Depression is a mood disorder marked by persistent sadness, loss of interest in daily activities, and physical and cognitive symptoms including changes in appetite and sleep patterns. Depression was assessed using the 21-item self-report Beck Depression Inventory-II (BDI-II) [22]. Responses were scored on a 0 (not at all) to 3 (severely) scale. Items were summed to create a single depression score, with higher scores indicating more severe depressive symptoms (*α* = 0.86). A score of 0–9 indicates minimal depression, 10–18 indicates mild depression, 19–29 indicates moderate depression, and 30–63 indicates severe depression.

##### Anxiety

Anxiety, a state of heightened worry, nervousness, or fear, was assessed using the 21-item self-report Beck Anxiety Inventory (BAI) [23]. Responses were scored on a 0 (not at all) to 3 (severely) scale. Items were summed to create a single anxiety score, with higher scores indicating more severe anxiety symptoms (*α* = 0.83). A score of 0–7 indicates minimal anxiety, 8–15 indicates mild anxiety, 16–25 indicates moderate anxiety, and 26–63 indicates severe anxiety.

##### Worry

Worry is a cognitive process involving repetitive thoughts about potential future threats or concerns. Worry was assessed using the 16-item self-report Penn State Worry Questionnaire (PSWQ) [24]. Responses were scored on a 0 (not at all typical) to 5 (very typical of me) scale. Items were averaged to create a single worry score ranging from 16 to 80, with higher scores indicating more worry (*α* = 0.65). An example item is, “My worries overwhelm me.”

#### Social functioning

##### Loneliness

Loneliness, a subjective feeling of social isolation or lack of meaningful connections, was assessed using the self-report Three-Item Loneliness Scale (TILS) [25]. Responses were scored on a 1 (hardly ever) to 3 (often) scale. Items were summed to create a single loneliness score ranging from 3 to 9, with higher scores indicating more loneliness (*α* = 0.82). An example item is, “How often do you feel that you lack companionship?”.

##### Social embeddedness

Social embeddedness is the extent to which an individual is integrated into social networks and experiences a sense of belonging within their community. The social embeddedness subscale of the Late-Life Changes in Social Relations Scale [26] was used to assess self-reported social embeddedness with family and friends. Responses were scored on a 1 (not at all) to 4 (more than six times) scale. Items were summed to create a single social embeddedness score ranging from 6 to 24, with higher scores indicating more embeddedness (*α* = 0.68). An example item is, “In the past two weeks, how often have you gone out to visit family?”.

#### Financial functioning

Financial functioning was assessed using the construct of instrumental risk taking, or willingness to engage in actions that involve uncertainty or potential harm. The 7-item instrumental risk taking subscale of the self-reported Stimulating-Instrumental Risk Inventory [27] was used. Responses were scored on a 1 (does not describe me at all) to 4 (describes me very well) scale. Items were averaged to create a single instrumental risk score ranging from 7 to 28, with higher scores indicating more instrumental risk taking (*α* = 0.76). An example item is, “If there is a big chance of profit I take even very high risks.”

#### Cardiopulmonary fitness

VO_2_peak was assessed at baseline for all participants and after the exercise intervention for older adults who completed the exercise intervention using an incremental graded exercise test to exhaustion with breath-by-breath gas collection (MGC Diagnostics Ultima, Saint Paul, MN) on a motorized treadmill (Full Vision Inc. Trackmaster, Newton, KS). We have reported elsewhere that participants in the MICT + IT condition experienced significantly greater increases in VO_2_max than those in the LICT condition [28]. While VO_2_max is defined as “the highest rate of oxygen uptake and utilization by the body during intense, maximal exercise” that is possible for an individual to achieve [29], VO_2_peak is the highest value of VO_2_ that an individual reaches on a specific test of high intensity exercise. VO_2_peak was used as an objective measure of fitness as it is not uncommon for untrained study subjects to tire before reaching the VO_2_ plateau requirements to measure VO_2_max. A modified Balke protocol was used [30], where treadmill speed remained constant and grade was increased by 2% every 2 min until exhaustion. A treadmill speed was selected to elicit approximately 70% of age predicted maximum heart rate and a rating of perceived exertion (RPE) of 13 (Borg; 6–20 scale) [31]. Heart rate was continuously monitored using a 12-lead electrocardiogram (EKG). VO_2_peak was calculated as the highest 30-s average during the test. Two participants unable to complete the VO_2_peak assessment on the treadmill due to an orthopedic limitation or a balance concern completed the test on a cycle ergometer (Lode Excalibur, Groningen, Netherlands). The tests began at a resistance of 0 Watts and increased by 20–25 Watts every 2 min until exhaustion.

### Statistical analysis

Tests of baseline equivalence were conducted using a series of independent samples *t*-tests. Impacts of exercise on each of the specified functional outcomes were assessed with mixed-design analyses of variance (ANOVAs) with one within-subjects factor (time: baseline, post-intervention) and one between-subjects factor (condition: LICT, MICT + IT) using the sample of older adults with baseline and post-intervention data (*n* = 161). Correlations between change in VO_2_peak and outcomes were also estimated. Power for the mixed ANOVAs was calculated post hoc. With an effect size of ƒ = 0.25, an estimated correlation of 0.50 among repeated measures, *α* = 0.05, two-tailed, power was estimated to be 0.99. With an effect size of ƒ = 0.10, an estimated correlation of 0.50 among repeated measures, *α* = 0.05, two-tailed, power was 0.71. Significant results were those with *p*-values < 0.05, while marginally significant results were those with *p*-values < 0.10.

### Funding

This work was supported by the National Institute on Aging R01AG43452 (NIA PI: Bryan) and the National Institute of Alcohol Abuse and Alcoholism F31AA029632 (NIAAA PI: Martin-Willett). Conclusions expressed in the manuscript are those of the authors and do not represent the opinions of the funding agencies.

## Results

### Sample characteristics

Two hundred eleven healthy older adults completed baseline measures of demographics, exercise behavior, and emotional, social, and financial functioning. There were no baseline differences between the LICT and MICT + IT conditions among participants who completed the trial (see Table 1), and no baseline differences emerged between those who completed the trial versus those who dropped out (see Appendix Table 5).^2^

**Table 1:** Baseline characteristics for older adult analysis sample, overall and by condition

		Full sample (*n* = 161)	LICT (*n* = 75)	MICT + IT (*n* = 86)	*p*-value
*Demographics*					
Age		67.33 (5.39)	67.55 (5.69)	67.14 (5.14)	*p* =.634
Sex	% Female	62.7	60.0	65.1	*p* =.503
Race/ethnicity	% Non-Hispanic White	90.1	90.7	89.5	*p* =.804
	% Hispanic or Latinx	4.3	4.0	4.7	
	% Asian	3.7	2.7	4.7	
	% Multiracial	0.0	0.0	0.0	
	% Non-Hispanic Black	0.0	0.0	0.0	
	% American Indian	0.6	1.3	0.0	
	% Not reported	1.2	1.3	1.1	
Education	% High school diploma or less	1.9	2.7	1.1	*p* =.723
	% Some college	13.8	16.0	11.7	
	% Bachelor’s degree	40.3	34.7	44.2	
	% Master’s degree or higher	44.0	45.3	41.9	
	% Not reported	1.2	1.3	1.1	
Employment	% Stay at home mom or dad	4.5	4.0	4.7	*p* =.053
	% Unemployed/disabled/retired	49.4	58.7	38.4	
	% Part-time (< 30 h/week)	21.8	14.7	26.7	
	% Full-time (≥ 30 h/week)	24.4	18.7	27.9	
	% Not reported	3.1	4.0	2.3	
Body mass index		27.30 (4.74)	27.45 (4.88)	27.17 (4.65)	*p* =.707
Exercise behavior		127.14 (378.78)	167.03 (520.37)	92.83 (182.34)	*p* =.218
*Social functioning*					
Loneliness		4.34 (1.55)	4.35 (1.49)	4.34 (1.60)	*p* =.954
Social embeddedness		13.74 (3.18)	13.61 (3.09)	13.85 (3.27)	*p* =.641
*Emotional functioning*					
Depression		7.30 (5.61)	7.68 (5.25)	6.97 (5.92)	*p* =.422
Anxiety		4.54 (4.48)	4.60 (4.36)	4.49 (4.61)	*p* =.875
Worry		2.37 (0.44)	2.36 (0.39)	2.39 (0.48)	*p* =.606
*Financial functioning*					
Instrumental risk taking		2.46 (0.52)	2.50 (0.50)	2.42 (0.54)	*p* =.349

For age, body mass index, exercise behavior, and the functioning variables, values are means with standard deviations in parentheses. LICT = low intensity continuous training; MICT + IT = moderate intensity continuous training + interval training. *p*-values reflect tests for equivalence across conditions (LICT vs. MICT + IT)

**Table 2:** Baseline associations for all study variables

Measure	1	2	3	4	5	6	7
1. Exercise behavior	___						
2. Loneliness	−.12	___					
3. Social embeddedness	.02	− **.31**	___				
4. Depression	−.08	**.52**	−.10	___			
5. Anxiety	−.06	**.39**	−.05	**.56**	___		
6. Worry	−.02	**.33**	−.05	**.42**	**.48**	___	
7. Instrumental risk taking	**.16**	−.10	.08	−.10	−.04	−.12	___

Correlations in bold are significant at the *p* <.05 level

### Exercise behavior and functioning at baseline

Participants reported an average of 15.5 min per week of moderate-to-vigorous intensity exercise at baseline as assessed by the PAR. Table 2 presents the zero-order baseline correlations for all study variables. Minutes of moderate-to-vigorous exercise per week shared a small significant correlation with instrumental risk taking but was not significantly associated with any of the social or emotional functioning measures at baseline.

### Impact of exercise on functioning

#### Emotional functioning

As can be seen in Table 3, participants across conditions exhibited a significant decrease in depression and a marginal increase in worry from baseline to post-intervention. There was also a significant, negative association between change in VO_2_peak and depression symptoms (*r* = − 0.188, *p* = 0.019) and a trend level association for anxiety (*r* = − 0.142, *p* = 0.077).

**Table 3:** Mixed ANOVA results for the impact of exercise on functioning among older adults (*n* = 161)

Measure	LICT		MICT+IT		Time		Condition		Time X Condition	
	Baseline	Post-intervention	Baseline	Post-intervention						
					*F*	*p*	*F*	*p*	*F*	*p*
Loneliness	4.35 (1.49)	4.58 (1.62)	4.32 (1.60)	4.08 (1.56)	0.001	.977	1.372	.243	5.986	**.016**
Social embeddedness	13.61 (3.09)	13.59 (3.10)	13.85 (3.31)	13.68 (2.90)	0.239	.626	0.129	.720	0.125	.724
Depression	7.68 (5.25)	6.71 (5.15)	6.93 (5.95)	4.89 (4.27)	22.166	**<.001**	2.863	.093	2.762	.099
Anxiety	4.60 (4.36)	4.60 (3.98)	4.47 (4.64)	3.96 (4.07)	0.800	.372	0.386	.536	0.800	.372
Worry	2.36 (0.39)	2.46 (0.43)	2.39 (0.48)	2.40 (0.44)	3.712	.056	0.016	.898	2.501	.116
Instrumental risk taking	2.50 (0.50)	2.41 (0.51)	2.41 (0.53)	2.34 (0.51)	6.718	**.010**	1.070	.303	0.092	.762

Values in the baseline and post-intervention columns are means with standard deviations in parentheses. LICT = low intensity continuous training; MICT+IT = moderate intensity continuous training + interval training

### Social functioning

**Fig. 2:**
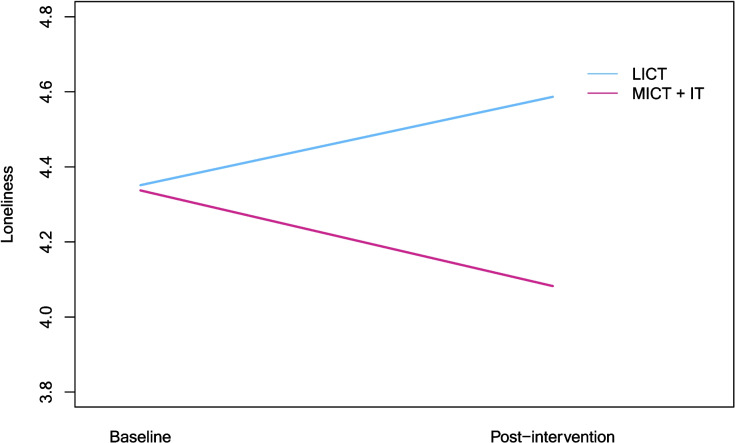
Interaction plot depicting change across time in loneliness by condition

We observed a significant timeXcondition interaction for loneliness (see Table 3). Simple effects tests indicated that loneliness was trending up for participants in the LICT condition across time [*F*(1, 157) = 2.73, *η*^2^ = 0.017, *p* = 0.100] but trending down for those in the MICT + IT condition [*F*(1, 157) = 3.29, *η*^2^ = 0.021, *p* = 0.072] (Fig. 2).

### Financial functioning

Finally, participants across conditions exhibited a significant decrease in instrumental risk taking from baseline to post-intervention (see Table 3). No significant effects of condition were observed.

## Discussion

This analysis sought to better understand the optimal intensity of exercise for improving the functional abilities that support instrumental activities of daily living that are critical for healthy cognitive aging in older adults [9]. This is among one of the very first studies to examine these direct relationships, adding much needed data to a topic area of high importance to the general public. Key national organizations are investing significant resources to help protect older adults from financial exploitation and other stressors (Money Management for Older Adults [32]; Consumer Financial Protection Bureau [33]), and the benefits of social inclusion and psychological wellbeing in aging are well established (E. S. [34, 35]).

In line with Tse et al.’s [12] conclusion that even low-intensity exercise interventions can reduce depression, participants across conditions in this study reported improvements in depressive symptoms. This is especially noteworthy considering they engaged in just 16 weeks of exercise, compared to other studies that have reported improvements in depression after longer exercise interventions (e.g., 6 months in Motl et al. [36]). We also observed a significant association between decreased depression symptoms and increases in VO_2_peak, suggesting cardiovascular fitness as a potential mechanism through which higher intensity exercise is linked to decreases in depression. On average, participants also had better posttest scores on a measure of instrumental risk taking. These improvements in emotional and financial functioning are particularly encouraging given our sample was relatively healthy at baseline and suggests increasing exercise at any intensity can be beneficial for older adults with varying health status. Our results also align with theoretical frameworks emphasizing the biopsychosocial benefits of physical activity [37] and suggest that structured exercise interventions can play a critical role in supporting key functional domains in older adults.

While we believe this to be the first study to explore the impact of physical activity on financial functioning, other research speaks to how exercise can improve a wide range of cognitive skills. As we previously reported benefits of exercise to executive function as measured with standardized cognitive tasks [28], we speculate it is perhaps via this route that financial functioning is improved. This is an emerging area of interdisciplinary research, with some early work suggesting executive function supports financial wellbeing even in the absence of financial content training [38], REF), while other more recent work is equivocal as to whether executive function plays a mediating or moderating role between knowledge and behaviors [39, 40]. Future work in this area could further investigate these relationships by making use of measures such as the Executive Personal Finance Scale [41] or the performance-based Direct Assessment of Functional Status-Extended Version [42] to better understand the underlying mechanisms. Overall, results encouragingly suggest exercise at any intensity level is beneficial to functional outcomes for healthy older adults.

Effects of exercise condition were less pronounced, similar to prior work suggesting relationships between exercise intensity, mood, and functional ability are not yet well understood. Participants assigned to the MICT + IT condition in our study showed lower levels of depression over time than those assigned to LICT, though the difference was only marginal. In an earlier study comparing effects of light, moderate, and vigorous exercise on symptoms of depression, all three exercise conditions had significant effects on depression compared to treatment-as-usual but were not significantly different from each other. Interestingly however, the difference in symptom reduction between the moderate and light intensity groups approached significance, with the light intensity group showing the greatest improvement of all groups [43]. Meanwhile, results suggesting participants in the LICT condition increased in loneliness while those in the MICT + IT condition decreased are fascinating when considered against other research in this area. Though the decrease in the MICT + IT condition is consistent with exercise intervention effects reported by other groups [44], the divergence by condition is still puzzling, particularly because all participants had equal opportunity for social interactions with research staff. Future studies could potentially vary the modality of exercise sessions (in person versus virtual) to manipulate social interaction while keeping intensity of exercise constant to better understand these effects.

Some limitations of our study should be considered. Our intervention did not include a time-matched non-exercise control group, and the reliability of the instrumental risk taking measure was suboptimal. Nonetheless, the fact that participants in the study demonstrated improvements in this area is very encouraging for future exploration, especially given the public health and economic significance of the ability of older adults to manage finances independently. In addition to this potential avenue for future work, it would also be advantageous to explore whether these findings extend to more diverse, less active, less healthy, or more sedentary populations who were precluded from participation in our study of healthy older adults. Relatedly, we were unable to assess whether the observed changes in emotional functioning were clinically meaningful as this was a non-clinical sample, with nearly all participants below the relevant clinical cutoffs at the start of the study.

## Conclusions

Broadly, findings from this study underscore the potential for tailored exercise programs to serve as scalable public health strategies for promoting psychological and financial well-being among older adults. Participants reported improvements in emotional and financial functioning after exercise training, and those in the higher intensity condition reported a greater decrease in loneliness and a slightly greater decrease in depression relative to those in the lower intensity condition. Similarly, greater increases in fitness, regardless of condition, were associated with greater decreases in depression symptoms and to some extent anxiety symptoms. This raises the possibility that there may be some benefits to higher intensity exercise that increases fitness for older adults. Future studies should seek to replicate these findings in less active and more diverse samples and across a wider range of exercise types and functional domains.

## Data Availability

The data that support the findings of this study are available from the senior author, [ADB], upon reasonable request.

## Appendix 1

Table 4

**Table 4:** Baseline characteristics by condition for older adult assigned sample (*n* = 211)

		LICT	MICT+IT	*p*-value
*Demographics*				
Sex	% Female	63.4	62.7	*p* =.923
Race	% White	95.0	91.8	*p* =.782
	% Asian	2.0	5.5	
	% American Indian	1.0	0.9	
	% More than one race	1.0	0.9	
	% Not reported	1.0	0.9	
Ethnicity	% Hispanic or Latino	5.0	4.5	*p* =.988
	% Not Hispanic or Latino	94.1	94.5	
	% Not reported	1.0	0.9	
Education	% High school diploma or less	3.0	1.8	*p* =.622
	% Some college	16.8	14.5	
	% Bachelor’s degree	31.7	40.9	
	% Master’s degree or higher	44.6	37.3	
	% Not reported	4.0	5.5	
Employment	% Stay at home mom or dad	4.0	4.5	*p* =.199
	% Unemployed, disabled, or retired	50.5	38.2	
	% Part-time (< 30 hours/week)	15.8	26.4	
	% Full-time (≥ 30 hours/week)	22.8	24.5	
	% Not reported	6.9	6.4	
Body mass index		27.92 (5.35)	27.24 (4.68)	*p* =.337
Exercise behavior		157.15 (470.91)	94.52 (178.01)	*p* =.200
*Social Functioning*				
Loneliness		4.35 (1.57)	4.43 (1.60)	*p* =.727
Social embeddedness		13.70 (3.14)	13.74 (3.09)	*p* =.929
*Emotional Functioning*				
Depression		7.69 (6.21)	7.52 (6.21)	*p* =.846
Anxiety		4.77 (4.76)	4.81 (4.72)	*p* =.947
Worry		2.35 (0.44)	2.41 (0.48)	*p* =.320
*Financial Functioning*				
Instrumental risk taking		2.47 (0.50)	2.42 (0.53)	*p* =.528

For body mass index, exercise behavior, and the functioning variables, values are means with standard deviations in parentheses. *LICT* = low intensity continuous training; *MICT+IT* = moderate intensity continuous training + interval training. *P*-values reflect tests for equivalence across conditions (LICT vs. MICT+IT)

## Appendix 2

Table 5

**Table 5:** Independent samples t-test results showing no differences in baseline exercise behavior and functioning between dropouts and completers

Measure	Attrition Status				t-test results	
	Dropouts		Completers			
	(*n* = 50)		(*n* = 161)			
	*M*	*SD*	*M*	*SD*	*t*	*p*
Exercise behavior	115.38	233.50	127.14	378.78	- 0.20	.840
Loneliness	4.57	1.73	4.34	1.55	0.83	.408
Social embeddedness	13.67	2.83	13.74	3.18	- 0.13	.893
Depression	8.79	8.02	7.30	5.61	1.39	.166
Anxiety	5.74	5.50	4.54	4.48	1.47	.144
Worry	2.41	0.55	2.37	0.44	0.50	.619
Instrumental risk taking	2.39	0.52	2.46	0.52	- 0.77	.441

## Footnotes

1 The delay between recruitment of participants and publication of results is primarily due to the extent of the overall project, which required careful analysis and dissemination of findings across multiple academic journal articles.

2 Also note that when using the full sample (*n* = 211), there were no baseline differences in demographics, exercise behavior, or functioning between those assigned to the LICT (*n* = 101) and MICT + IT (*n* = 110) conditions (see Appendix Table 4).
